# Morphological correlates of distal fibular morphology with locomotion in great apes, humans, and *Australopithecus afarensis*

**DOI:** 10.1002/ajpa.24507

**Published:** 2022-03-17

**Authors:** Damiano Marchi, Andreas Rimoldi, Daniel García‐Martínez, Markus Bastir

**Affiliations:** ^1^ Department of Biology University of Pisa Pisa; ^2^ Centre for the Exploration of the Deep Human Journey University of the Witwatersrand Wits; ^3^ Centro Nacional de Investigación sobre la Evolución Humana (CENIEH) Burgos Spain; ^4^ Paleoanthropology Group Museo Nacional de Ciencias Naturales (MNCN‐CSIC) Madrid Spain

**Keywords:** 3D geometric morphometrics, arboreal locomotion, bipedal locomotion, fibula, functional morphology

## Abstract

**Objectives:**

Recent studies highlighted the importance of the fibula to further our understanding of locomotor adaptations in fossil hominins. In this study, we present a three‐dimensional geometric morphometric (3D‐GM) investigation of the distal fibula in extant hominids and *Australopithecus afarensis* with the aim of pointing out morphological correlations to arboreal behavior.

**Methods:**

Three‐dimensional surface meshes of the distal fibula were obtained using computer tomography for 40 extant hominid specimens and laser scanner for five *A. afarensis* specimens. Distal fibula morphology was quantified positioning 11 fixed landmarks, 40 curve semilandmarks, and 20 surface landmarks on each specimen. A generalized Procrustes analysis (GPA) was carried out on all landmark coordinates followed by Procrustes ANOVA. Principal component analysis (PCA) was performed on the GPA‐aligned shape coordinates. Kruskal‐Wallis tests and Mann–Whitney test were performed on scores along PCs.

**Results:**

Great apes are characterized by a shorter subcutaneous triangular surface (STS), more downward facing fibulotalar articular facets, more anteriorly facing lateral malleolus and wider/deeper malleolar fossa than humans. Within great apes, orangutans are characterized by more medially facing fibulotalar articular facets. *Australopithecus afarensis* shows a unique distal fibular morphology with several traits that are generally associated more to arboreality and less to bipedalism such as a short STS, a more anteriorly facing, laterally pointing malleolus and deeper and larger malleolar fossa.

**Conclusions:**

The distal fibula morphology is indicative of locomotor patterns within extant hominids. The 3D‐GM method presented here can be successfully used to further our understanding of arboreal adaptations in fossil hominins.

## Graphical abstract

Distal fibula morphology of extant hominids and *Australopithecus afarensis*.

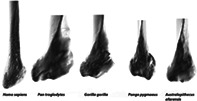

## 1 INTRODUCTION

The traits that uniquely characterize hominins when compared to the other primates are their upright posture and obligate bipedal locomotion. However, there is still no consensus on the type of bipedalism of early australopiths, in particular concerning the amount of time spent in the arboreal environment (Berge, 1994; Crompton et al., 2008; Harcourt‐Smith & Aiello, 2004; Latimer & Lovejoy, 1990a, 1990b; Latimer et al., 1987; Stern, 2000; Stern & Susman, 1983; Ward, 2013). Although most of the studies addressing this topic have focused on femur and tibia, or on the feet, recent studies have shown that the morphology of the fibula, in particular its diaphyseal strength (as expressed by cross‐sectional geometric properties) can also further our understanding of the degree of arborealism of early hominins (Marchi, 2007; Marchi, 2015a; Marchi et al., 2019).

Within mammals, it is generally acknowledged that the fibula morphology and mobility vary according to their locomotor behaviors (Barnett & Napier, 1953). For example, jumping mammals show a fibula partially fused (i.e., partially mobile) to the tibia distally, while fossorial and swimming mammals show a fibula proximally and distally fused (i.e., immobile fibula) to the tibia. An immobile fibula is also found in mammals specialized in running, like ungulates. The rarest mobile fibula is present in carnivores, such as bears and felids, and in primates including modern humans (hereafter humans): this fibular morphology is associated with the wide range of dorsiflexion/plantarflexion and eversion/inversion of the ankle joint common in carnivores and primates during their habitual locomotion (Barnett & Napier, 1953). It has also been observed that different fibular morphologies correspond to characteristic size proportions between the tibia and the fibula: the fibula is relatively (to the tibia) more robust in fossorial and swimming mammals, intermediate in mammals adapted to move over uneven terrain such as primates and carnivores, and less robust in more saltatorial mammals (Barnett & Napier, 1953). Relevant to the present investigation, the intermediate robusticity of the nonhuman primate fibula is associated with the high mobility of it, in turn related to the high degree of ankle mobility which is generated by the accentuated dorsiflexion and inversion of the foot generated during locomotion in the arboreal setting where primates mainly move (Barnett & Napier, 1953; Carleton, 1941; DeSilva, 2009; Latimer et al., 1987; Stern & Susman, 1983; Walmsley, 1918).

Of particular relevance to the present study is the observation that the relative increase in fibular diaphyseal strength among more arboreal hominids is related to foot and leg positioning during arboreal behavior (Marchi et al., 2019). During vertical climbing, for example, the foot of nonhuman hominids is subjected to greater dorsiflexion of the ankle–which has been hypothesized to be related with greater load on the fibula (Barnett & Napier, 1953)–to bring the body close to the substrate, while during vertical climbing and walking in trees, the foot is subjected to greater inversion at the ankle and more abduction of the subtalar joint than in terrestrial locomotion (DeSilva, 2009; Holowka et al., 2017). During climbing/clambering, nonhuman hominids bear the hip abducted and flexed and the thigh abducted (DeSilva, 2009; Wunderlich & Ischinger, 2017) resulting in a more laterally displaced knee (Isler, 2005). This will in turn increase the mediolateral bending loads in the leg and the importance of the lateral bone, that is, the fibula, in preventing excessive mediolateral strains of the leg (Wang et al., 1996). Analyses of fibula/tibia diaphyseal strength ratios in several catarrhines (Marchi, 2007, 2015a) indeed demonstrated that the more arboreal species were characterized by relatively more robust fibulae than the more terrestrial ones. Though the primate leg is subjected to mediolateral bending loads both in arboreal and terrestrial locomotion, the abducted limb positioning born during arboreal locomotion by more arboreal primates would be expected to increase mediolateral bending loads on the leg compared to terrestrial moving, as suggested by the greater fibula/tibia diaphyseal strength ratios in the mediolateral plane than in the anteroposterior plane found in hominids involved in high degree of arboreal behavior (Marchi et al., 2019).

Although fibula/tibia diaphyseal strength ratios are good indicators of arboreal locomotion in extant hominids (Marchi, 2007, 2015a), and therefore can be used to infer the degree of arborealism in fossil hominins (Marchi et al., 2019), its use is restricted due to the scarcity of associated tibiae and fibulae in the fossil record. The only early australopithecine associated tibiae and fibulae available till recently were those from A.L. 288–1 (*Australopithecus afarensis*, Johanson & Taieb, 1976), although the partial distal fibula is too fragmentary for the application of the method. Recently, associated tibiae and fibulae have been described in South Africa in association with the StW 573 *Australopithecus* sp. skeleton (Heaton et al., 2019) but a biomechanical study of the leg has not been provided yet. However, besides the A.L. 288–1 distal fibula (A.L. 288‐1 at), four others *A. afarensis* isolated (i.e., not associated with the tibia) distal fibulae in good state of preservation are present in the fossil record from Hadar: A.L. 333‐9a, A.L. 333‐9b, A.L. 333–85, A.L. 333w‐37 (Lovejoy et al., 1982).

Early investigation of the five *A. afarensis* distal fibulae found several differences between *A. afarensis* and humans (Stern & Susman, 1983). The proximal border of the *A. afarensis* fibulotalar articular facet runs a course that is oblique to the longitudinal axis of the fibula, similar to what is observed in great apes. Moreover, the proximal portion of the *A. afarensis* fibulotalar articular facet faces more inferiorly than the human one, looking more similar to that of great apes. Stern and Susman (1983) also described broader peroneal grooves for *A. afarensis* than for humans, which they interpreted as indicating more powerful peroneal muscles in the fossil species. However, the authors noted that the lateral malleolus of *A. afarensis* is less anteriorly oriented than in great apes, though the subcutaneous triangular surface (STS) is not as laterally oriented as in humans.

The interpretation of the distal fibula morphology provided by Stern and Susman (1983) was challenged by Latimer et al. (1987) based on their observation that the congruence of the talofibular joint could not be assessed with confidence from the fibula alone. Further, a recent study performed on the tibia (DeSilva, 2009) suggested that the distal anatomy of the bone of *A. afarensis* did not show evidence of neither loading of the ankle in dorsiflexion nor of inverted foot set, therefore denoting inability of *A. afarensis* to climb in an ape‐like manner. However, a more recent study (Venkataraman et al., 2013) found a high degree of ankle dorsiflexion in modern hunter‐gatherers involved in regular tree climbing which was not associated with any osteological signal of hyperdorsiflexion of the foot. This last evidence would suggest a more complex than expected relationship between hominin climbing behavior and ankle skeletal features.

Recently, a quantitative analysis of the distal fibula morphology in great apes, humans, and *A. afarensis* was performed with the aim of elucidating the morphologies in this region of the leg in relation to arboreal behavior (Marchi, 2015b). The study took into consideration linear measurements of the fibulotalar articular facets, the angles formed by the fibulotalar articular facets and the longitudinal axis of the fibula, and the angle between the proximal fibulotalar articular facet and the STS. Results revealed that: (1) more terrestrial hominids have larger (relative to the proximal) distal fibulotalar articular facet than more arboreal ones; (2) great apes have smaller distal fibulotalar articular facet area and more downward facing fibulotalar articular facets than humans; (3) *Pan* and *Gorilla* have a more anteriorly facing lateral malleoli than humans and *Pongo*; and (4) australopiths display some traits consistent with modern human‐like bipedalism, such as a more laterally facing lateral malleoli, in association with more ape‐like traits, such as more downward oriented fibulotalar articular facets, consistent with *A. afarensis* being a bipedal terrestrial hominin adapted for some form of climbing, in agreement with other studies on postcranial australopith morphology (Stern & Susman, 1983).

One limitation of Marchi's (2015b) study (as of other previous studies involved in this region of the leg) was that linear measurements were used and therefore subtle differences in the shape of the distal fibulotalar articulation could not be detected. For example, fibulotalar articular facet areas in Marchi's (2015b) study were calculated using geometric formulae derived from the average shape of the facets. For example, given that the proximal fibulotalar articular facet in *Pan* shows on average an elliptical shape (see Marchi, 2015b: Figure 2), the maximum and minimum linear breadths of the facet were used in the ellipse area formula for calculating the facet area. Estimation of articular surfaces by approximation using geometric formulae has been done for other bones of the postcranial skeleton (humerus, femur, tibia and metatarsals) with reasonable results (Marchi, 2010; Rafferty & Ruff, 1994; Ruff, 2002). However, as stated in each of these studies, articular facets very rarely, if ever, can be represented accurately by geometric shapes and therefore the areas used for the analyses are approximation of the real area of the articular facets.

Another limitation of Marchi's (2015b) study was the lack of consideration of the STS's morphology. The STS is the area in the disto‐lateral portion of the fibula which is not covered by muscles and is lined cranially and posteriorly by the tendons of the mm. *peroneus longus* and *peroneus brevis* (Aiello & Dean, 2002). Peroneal muscles are recruited in chimpanzees mainly during the support phase of locomotion on either vertical or horizontal trunks in the action of everting the foot (Stern & Sussman, 1983), regulating the transfer of weight on the medial part of the inverted foot during climbing. In humans, the mm. *peroneus longus* and *peroneus brevis* are recruited in the second half of stance phase during normal level walking (Stern & Susman, 1983). A recent work (Bavdek et al., 2018), where human subjects walked on a flat surface and on a medial incline ramp, showed that the electromyographic (EMG) activities of peroneal muscles increased when walking on the medial incline ramp, therefore everting the inverted foot.

**FIGURE 1:**
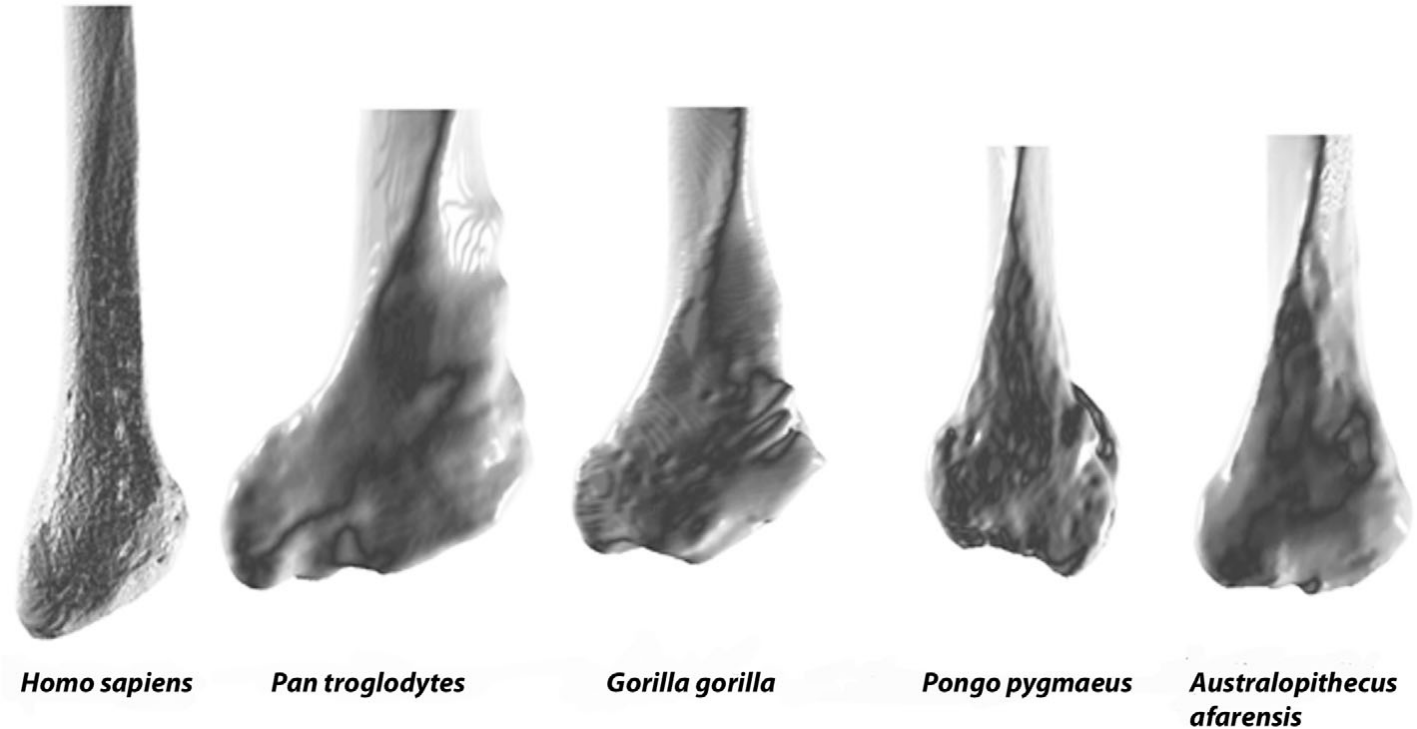
Example of average distal fibula morphology (lateral view) of the extant species and of *Australopithecus afarensis*. *Homo sapiens*: Raymond A. Dart collection, University of the Witwatersrand, South Africa, specimen A98; *Pan troglodytes*: Schultz collection, University of Zurich Irchel, Switzerland, specimen number 220; *Gorilla gorilla*: Schultz collection, University of Zurich Irchel, Switzerland, specimen number 12; *Pongo pygmaeus*: Primate collection, Zurich, Switzerland, specimen number 8685*; Australopithecus afarensis*: Cast from the University of the Witwatersrand, South Africa, specimen number A.L. 288‐1 at

While Stern and Susman (1983) provided qualitative evidence of the different STS orientation in great apes and humans and its relationship with the different development of peroneal muscles in the two groups, a quantitative analysis of the STS orientation has never been attempted. In humans, the STS is more cranially elongated than in great apes (Figure 1; see also Aiello & Dean, 2002: Figure 22.21 and Swindler & Wood, 1973: Plate 147). Aiello and Dean (2002) described this structure as forming a blunt and laterally convex curve in humans as opposed to a sharper and laterally concave curve in great apes. The craniocaudally shorter STS of great apes is associated with longer bellies and shorter tendons (relative to the muscle total unit) of the peroneal muscles, while the craniocaudally longer STS of humans is associated with shorter bellies and longer tendons (Marchi et al., 2018; Payne et al., 2006; Raven, 1950; Swindler & Wood, 1973). More proximally distributed muscle mass in the limbs, and therefore longer tendons, which are well suited to economical force development (short muscle fascicles) and elastic energy saving (long tendons), are generally found in those animals that need to save mass in the distal part of their limbs to allow higher efficiency and speed in the movement of the distal part of their limb (for example, unguligrades, running birds and wallabies, Brown & Yalden, 1973; Biewener & Roberts, 2000; Payne et al., 2005). Longer muscle bellies extending closer to the distal part of the limb favor the ability to produce more power and control on the distal part of the limb (Biewener, 2016). Human bipedalism requires a condition similar to that of running animals, in order to move the distal part of the lower limb efficiently at high speed for long periods in bipedal locomotion (Bramble & Lieberman, 2004). Great apes, instead, need strong force and control in their lower extremities (i.e., feet) to grasp branches and move in an arboreal environment (Morton, 1924; Payne et al., 2006; Vereecke et al., 2005). The STS morphology appears therefore correlated to peroneal muscle morphology in extant hominids and its quantification could be of interest in an evolutionary perspective when attempting to evaluate the degree of arborealism in fossil hominins.

To capture the morphology of the distal fibula articulation, here we apply a three‐dimensional geometric morphometric (3D‐GM) approach to the study of the distal fibular portion of humans and great apes (*Homo, Pan*, *Gorilla* and *Pongo*) and *A. afarensis* with the aim of better understanding the correlates of distal fibular shape with arboreal behavior in extant hominids and fossil hominins. Many recent studies have used the 3D‐GM approach to address functional morphological questions (e.g., Almécija et al., 2013; Arias‐Martorell et al., 2012; De Groote, 2011; Fernández et al., 2018; Marchi et al., 2017; Marchi et al., 2019; Rein et al., 2017; Sorrentino et al., 2021). The 3D‐GM method will provide more information about the shape variability that can inform about functional interpretation of fossil hominin distal fibula morphology.

Building upon previous studies on the subject, we hypothesize that:

- The shape of the distal fibula in extant hominids will significantly differ between humans and great apes (Marchi, 2015b). In particular, we expect to find: (1) more anteriorly facing lateral malleolus in great apes than in humans, reflecting the more powerful peroneal muscles in great apes than in humans (Stern & Susman, 1983), and (2) fibulotalar articular facets more downward facing in great apes than in humans, reflecting the higher mobility of the ankle joint in great apes than in humans (e.g. Barnett & Napier, 1953; DeSilva, 2009; Latimer et al., 1987; Stern & Susman, 1983);
- The STS will be craniocaudally longer in humans than in great apes, reflecting the need for lighter distal lower limb extremities in bipedal humans compared to the grasping foot of arboreal great apes (Morton, 1924; Payne et al., 2006).

*Australopithecus afarensis* has been alternatively modeled as obligate bipedal for whom arboreality was adaptively insignificant (e.g., Crompton et al., 1998; Latimer et al., 1987; Sayers & Lovejoy, 2008) or as primarily bipedal but retaining a significant adaptation to arboreality (e.g., Stern and Sussman, 1983; Senut, 1988; Duncan, 1994). It is beyond the scope of this paper to enter the argument concerning early hominin locomotion. However, we can make predictions for shape results based on the two competing models. If the obligate bipedal model was true than we predict to find a distal fibula morphology with a laterally facing lateral malleolus, fibulotalar articular facets medially facing, and an STS craniocaudally long, more similar to humans than to great apes. If the model that includes significant adaptation to arboreality (and therefore climbing behavior) was true, then we predict to find in *A. afarensis* an anteriorly facing lateral malleolus, fibulotalar articular facets downward facing, and an STS craniocaudally short.

## 2 MATERIALS AND METHODS

### 2.1 The sample

The extant sample includes the fibula of recent *Homo sapiens* (*n* = 16), *Gorilla gorilla* (*n* = 6), *Pan troglodytes* (*n* = 9), *Pongo pygmaeus* (*n* = 8) and *Pongo abelii* (*n* = 1) (Table 1). For each individual the right fibula was used and, when the right was not available, the left was taken. Only adult, non‐pathological individuals were included in this study and every specimen was carefully evaluated for a completely fused epiphysis in the fibula and other postcranial material when available. Though small differences in positional behaviors among the two species of orangutans are present the kind of arboreal locomotion employed by the two species does not vary much (Cant, 1987; Manduell et al., 2012). Therefore, for the purpose of this study *P. pygmaeus* and *P. abelii* were pooled in the same sample, and species level differences were not investigated in the current study. The fossil sample includes the left distal fibula from *A. afarensis* A.L. 288‐1 at and the right distal fibulae from *A. afarensis* A.L. 333‐9a, A.L. 333‐9b, A.L. 333‐85 and A.L. 333‐w37 (SOM Figure S1; Table 1).

**TABLE 1:** Sample composition

Taxon	*n*/fossil ID		Sex			Side	
		Institution	Male	Female	Unknown	Right	Left
Extant							
*Homo sapiens*	16	DartColl	7	9		16	—
*Pan troglodytes*	9	SCZ, PCZ, SZCM	6	2	1	8	1
*Gorilla gorilla*	6	SCZ, PCZ, SZCM	4	2		3	3
*Pongo pygmaeus*	8	SCZ, PCZ, SZCM,	3	5		6	2
*Pongo abelii*	1	PCZ		1		—	1
Fossils							
*Australopithecus afarensis*	A.L. 288‐1 at	WITS				1	—
*Australopithecus afarensis*	A.L. 333‐9a	WITS				1	—
*Australopithecus afarensis*	A.L. 333‐9b	WITS				—	1
*Australopithecus afarensis*	A.L. 333–85	WITS				—	1
*Australopithecus afarensis*	A.L. 333‐w37	WITS				—	1

Abbreviations: DartColl: Raymond A. Dart Collection, Department of Anatomical Sciences, University of the Witwatersrand, Johannesburg, South Africa; PCZ = Primate Collection, University of Zürich Irchel, Zurich, Switzerland; SCZ, Schultz Collection University of Zürich Irchel, Zurich, Switzerland; SZCM, State Zoological Collection, Münich, Germany; WITS, Evolutionary Studies Institute, University of the Witwatersrand, Johannesburg, South Africa.

Three‐dimensional surface meshes of the distal fibula of the extant sample were obtained using Computer Tomography (CT) scanning. All meshes were obtained using Avizo 8 software (Visualization Sciences Group, Merignac, France) selecting the *Surface view* module, then in the *more options* button in the *Draw style* port selected *Create surface*. *Gorilla*, *Pan* and *Pongo* meshes were obtained from medical CT scans performed at the Munich Institute for Radiology Ludwig Maximilian University (Munich, Germany) on a GE Discovery CT750 HD medical CT scanner (slice thickness 0.625 mm, slice increment 0.3 mm, voltage 120 kV, X‐ray tube current 99 mA, reconstructing algorithm bone, pixel size 460 μm) and at the University Hospital of Zurich (Zurich, Switzerland) on a Siemens Somaton Definition Flash (slice thickness 0.6 mm, slice increment 0:3 mm, voltage 120 kV, current 19 mA, reconstructing algorithm bone, pixel size 600 μm). Human individuals were scanned at the Microfocus X‐Ray Computed Tomography facility of the University of Witwatersrand (Johannesburg, South Africa) on a Nikon Metrology XTH 225/320 LC (Voltage 70 kV, current 120 μA, no filter used, pixel size 120 μm). Surface models of the research quality fossil casts present at the Evolutionary Studies Institute and Centre for Excellence in PalaeoSciences (University of Witwatersrand, South Africa) were made using a NextEngine laser scanner (pixel size 125 μm). Surface models obtained by CT scans and laser scans are comparable (Slizewski et al., 2010; Kanawati et al., 2020), therefore fossil and living specimens can be compared in this study.

### 2.2 3D geometric morphometrics

**FIGURE 2:**
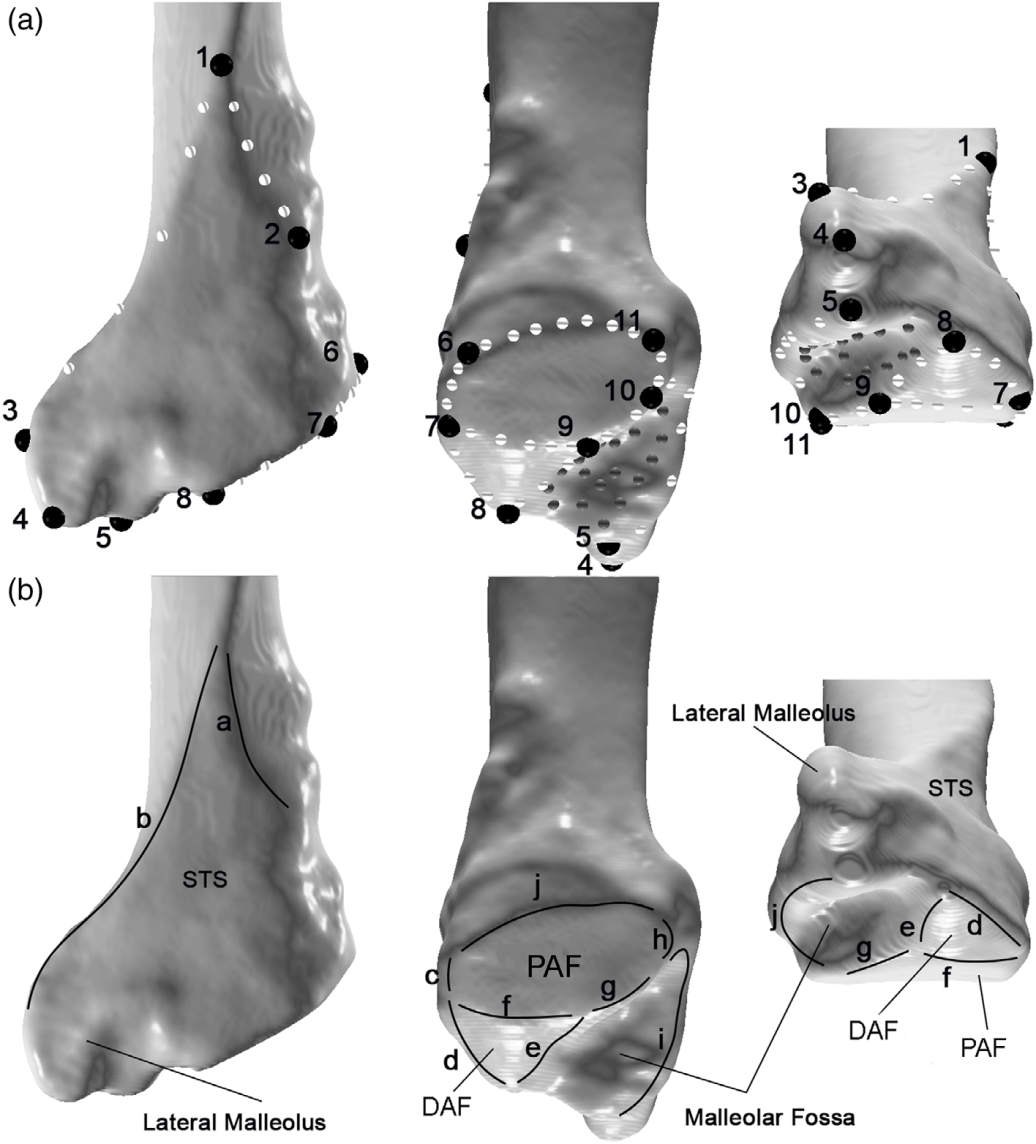
(a) Landmark configuration on a *Gorilla* right fibula. Left, lateral view, center, posterior view and right, distolateral view. Black large dots are fixed landmarks, small white dots are curve semilandmarks and small gray dots are surface semilandmarks. Definition of numbered semilandmarks is in Table 2; (b) Curves definition on a *Gorilla* left fibula. Left, lateral view, center, posterior view, and right, distal view. Definition of curves is in Table 3. DAF, distal fibulotalar articular facet; PAF, proximal fibulotalar articular facet; STS, subcutaneous triangular surface

To quantify the morphology of the distal fibula we positioned 71 landmarks on each specimen (Figure 2, Tables 2 and 3) using the software Viewbox4 (Dhal software version 4.0.1.7, http://www.dhal.com/viewboxindex.htm; Bastir et al., 2019). Because of uncertainty in terms of their locations along the distal fibulae, semilandmarks were slid along their corresponding curves with respect to the fixed landmarks to minimize bending energy, first between each specimen and the template (first specimen) and after that, a second time against the sample average configuration (Bastir et al., 2013, 2017; Mitteroecker & Gunz, 2009).

**TABLE 2:** Definition of the 11 fixed landmarks

Landmark	Definition
1	Point where the anterior ridge (anterolateral in humans) divides into two ridges
2	Most medial point of the medial border of the STS
3	Most lateral point of the lateral border of the STS
4	Most distal point of the lateral malleolus in anterior view
5	Most distal point of the posterior malleolar fossa border
6	Most anterior point on the anterior border of PAF
7	Point between the anterior border of PAF and the anterior border of DAF
8	Most distal point of DAF
9	Most proximal point of DAF posterior border
10	Point where the posterior malleolar fossa border meets the PAF border
11	Most posterior point of the proximal border of the PAF

Abbreviations: DAF, distal fibulotalar articular facet; PAF, proximal fibulotalar articular facet; STS, subcutaneous triangular surface. See Figure 2 for illustration of each articular surface.

**TABLE 3:** Definition of the 10 curves

Curve	Number of semilandmarks	Definition
a	5	Medial border of the STS
b	5	Lateral border of the STS
c	3	Anterior border of the FiTal1Ar
d	3	Anterior border of the FiTal2Ar
e	3	Posterior border of the FiTal2Ar
f	5	Border between FiTal1Ar and FiTal2Ar
g	2	Distal section of the FiTal1Ar posterior border, defined by L9 and L10
h	2	Proximal section of the FiTal1Ar posterior border, defined by L10 and L11
i	5	Posterior border of malleolar fossa
j	7	Proximal border of FiTal1Ar, defined by L6 and L11.

*Note*: L9, L10, and L11 stands for fixed landmark 9, 10 and 11, respectively. See Figure 2 for illustration of each articular surface and position of each fixed landamark.

Abbreviations: STS, subcutaneous triangular surface; FiTal1Ar, proximal fibulotalar articulation; FiTal2Ar, distal fibulotalar articulation.

The landmark configuration consisted of 11 operator‐defined fixed landmarks (Table 2), 40 curve semilandmarks (Table 3), and 20 surface semilandmarks on the articular surface (Figure 2). This configuration describes the whole morphology of the distal fibula, including the STS, the lateral malleolar region and the fibulotalar articulations. Four out of five of the *A. afarensis* specimens (A.L. 333‐9a, A.L. 333‐9b, A.L. 333‐85 and A.L. 333‐w37) were missing the most proximal region of the STS (SOM Figure S1), hence a geometric reference‐based estimation of the missing landmarks (Bastir et al., 2019; Gunz et al., 2009) was performed in Viewbox 4, using the most complete individual, A.L. 288‐1 at, as reference specimen. This reference‐based approach takes advantage of the actually preserved morphology of each of the four fossils and uses the complete A.L. 288‐lat conspecific specimen as reference geometry to transform it into the configuration of each of the incomplete fossil, thereby calculating the values of the missing structure. Of the 71 landmarks positioned on each specimen, only the fixed landmark N. 1 (Table 2) was missing in each fossil, therefore the part to be estimated with this approach was minimal. Several studies have demonstrated the strength of the results obtained through such estimations (Gunz et al., 2009; Bastir et al., 2021; García‐Martinez et al., 2020).

A generalized Procrustes analysis (GPA, Gower, 1975) was carried out on all landmark coordinates and both curve and surface semilandmarks were slid to minimize bending energy (Rohlf, 2010). We performed a Procrustes ANOVA (Collyer et al., 2015) to assess the existence of a significant shape difference among the different groups, then principal component analysis (PCA) was performed on the GPA‐aligned shape coordinates to visualize and quantify shape changes. To avoid the problem of non‐normally distributed data, a Kruskal‐Wallis tests by ranks were performed on scores along principal components (PCs), testing for significant differences among extant genera and *A. afarensis*, followed by a Mann–Whitney test to explore pairwise comparisons. Scatterplots and box‐and‐whisker plots were used to graphically represent data distributions.

Prior to the statistical analysis, we tested the repeatability of our landmark configurations using a multivariate version of the Levene's test called Anderson test (Anderson, 2006). Following Galletta et al. (2019), we repeated six times the landmarks placing procedure on a single specimen for every genera, testing the hypothesis that lower variance due to relative clustering should verify the repeatability of landmarks. We used the Anderson test on the first two PCs to assess the heteroscedascity between the repeated measures and the rest of the sample of the same genus. We tested for allometric signal in the data in two ways: a) by a traditional allometric analysis of fibular shape on centroid size; b) by Procrustes ANOVA (Collyer et al., 2015) using centroid size (CS), a proxy of body size, as a covariate. Further, a Procrustes regression analysis of shape on size was carried out in a phylogenetic framework using the procD.pgls function of the package *geomorph* in R (Adams & Otárola‐Castillo, 2013). The phylogenetic trees used for the analysis were built using estimated divergence times published on timetree.com (Kumar et al., 2017).

All statistical analyses were performed in R environment (R Core Team, 2013), using functions of the package *geomorph* v 3.1.2 (Adams & Otárola‐Castillo, 2013), and *vegan* v 2.5‐3 (Oksanen et al., 2018).

## 3 RESULTS

### 3.1 Repeatability

Repeated measure in the morphospace cluster together and are easily recognizable from the rest of the sample, for all the taxa examined (SOM Figure S2). Statistical testing supports the separation observed in the graphs (*p* <0.05). Therefore, we can conclude that landmarks positioning is repeatable for the purpose of this study.

### 3.2 Allometric analysis

**FIGURE 3:**
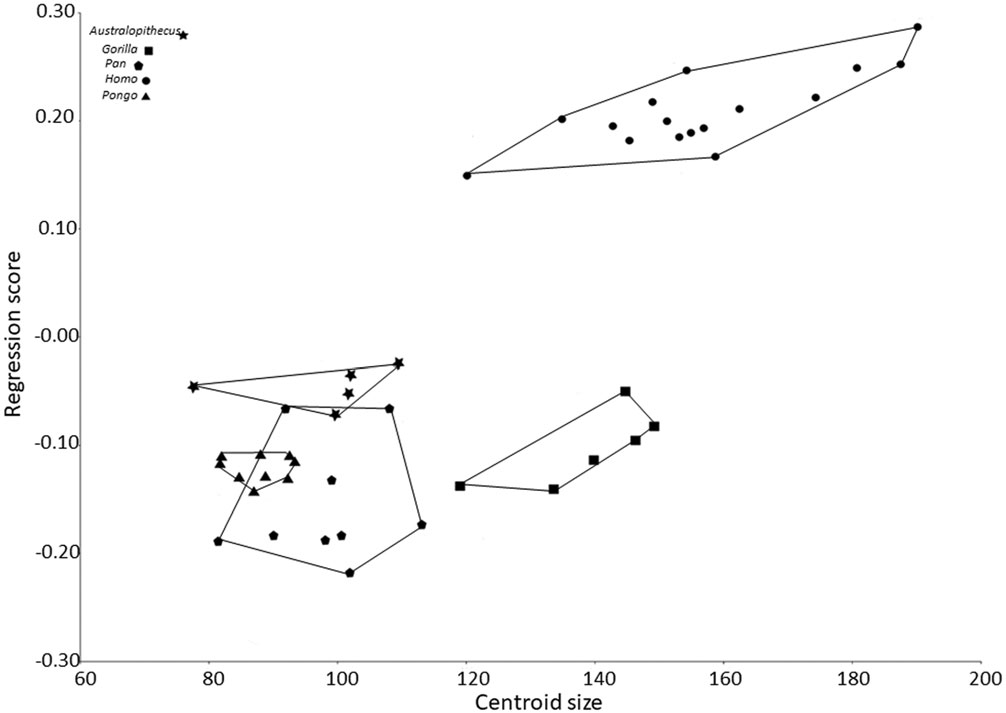
Scatterplot of centroid size versus regression scores of extant samples (*Homo, Pan, Gorilla* and *Pongo*) and fossil specimens A.L. 288‐1 at, A.L. 333‐9a, A.L. 333‐9b, A.L. 333–85, A.L. 333w‐37 (labeled ‘*Australopithecus*’ in figure)

The regression of fibular shape on centroid size (Figure 3) shows a positive correlation between shape and size (R^2^ = 44%, *p* < 0.01). However, it is important to note that part of this relation is a statistical artifact: our groups differ both in size and in shape, but not all these shape differences should be explained by allometric size differences, as indicated in the figure. It would be more appropriate to state that there are two different trends: towards positive regression scores there is clearly an influence of bipedalism (humans and australopiths plot more positively, while the rest plots more negatively). *Australopithecus* is intermediate between bipedal humans and arboreal apes.

Results of the Procrustes ANOVA indicates a significant effect of shape for every subsample of specimen examined (*p* < 0.001), however the phylogenetic comparative analysis returns a non‐significant *p* value (> 0.05), indicating that size does not significantly influence the shape of the distal fibula, when analyzed in a phylogenetic framework. Overall, for the aims of this study, the results suggest that we can exclude size as an important factor in the interspecific variation of the shape.

### 3.3 Procrustes ANOVA, PCA, Kruskal‐Wallis, and pairwise Mann–Whitney's test

Results of the Procrustes ANOVA (Table 4) indicates a significant difference of shape among the groups involved (*p* < 0.01). The first three PCs in the PCA account for more than 79% of total variance. PC1 explain 68.8% of variance, PC2 7.0% and PC3 4.1%. Principal Component 4 and beyond are not statistically significant (*p* > 0.05), therefore are not taken into consideration. The Kruskal‐Wallis test shows that the group separation along PC1 (*p* < 0.01), PC2 (*p* < 0.01) and PC3 (*p* < 0.01) is statistically significant (Table 5).

**TABLE 4:** Procrustes ANOVA results. Permutation model: Randomized of null model residuals. Number of permutations: 10,000

	Df	R^2^	*p* value
Species	4	0.76544	**<0.001**
Residuals	40	0.23456	

*Note*: Estimation method: Ordinary least squares.

**TABLE 5:** Kruskal‐Wallis test results for the first three principal component (PCs)

Principal component	Chi‐squared	*p* value
PC1	36.3	**<0.001**
PC2	33.8	**<0.001**
PC3	16.4	**0.002**

*Note*: Statistically significant *p* values scores are in bold.

**FIGURE 4:**
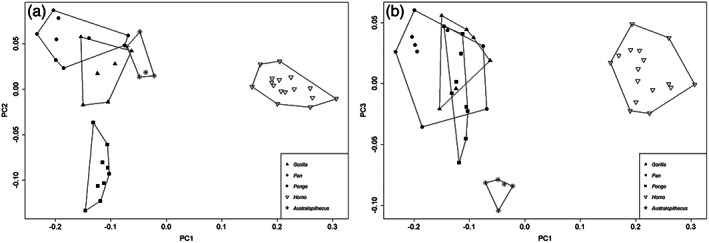
Scatterplot of (a) second versus first principal component (PC2 vs. PC1) scores and (B) third versus first principal component (PC3 vs. PC1) scores (on the right) of extant samples (*Homo*, *Pan*, *Gorilla*, and *Pongo*) and fossil specimens A.L. 288‐1 at, A.L. 333‐9a, A.L. 333‐9b, A.L. 333–85, A.L. 333w‐37 (labeled ‘*Australopithecus’* in figure)

A 3D scatterplot of PC1, PC2, and PC3 clearly shows the separation between *Pongo*, African great apes, humans and *A. afarensis* (SOM Figure S3). A bivariate scatterplot of PC1 against PC2 (Figure 4a) separates humans from great apes and within great apes *Pongo* from African apes. *Australopithecus afarensis* specimens fall in the same quadrant as African great apes, marginally overlapping with *Gorilla*'s morphospace. Along PC1, humans are significantly different from all great apes (Table 6) and great apes are not significantly different from each other. *Australopithecus afarensis* is significantly different from all extant genera. Along PC2, *Pongo* is significantly different from every other genus (Table 7). African great apes are not significantly different from each other, and humans are significantly different from every other group but *Gorilla* (Table 7). *Australopithecus afarensis* is significantly different from *Pongo* and humans but not from African great apes. A bivariate scatterplot of PC1 against PC3 (Figure 4b) successfully separate humans (along PC1) from great apes, which greatly overlap in their distributions. *Australopithecus afarensis* occupies the left lower quadrant closer to great apes than to humans, but completely separated from them. Along PC3 all extant genera are not significantly different from each other while *A. afarensis* is significantly different from all extant genera (Table 8).

**TABLE 6:** Pairwise Wilcoxon test on principal component 1 scores

	Gorilla	Pan	Homo	A. Afarensis
*Pongo*	0.955	0.188	**<0.001**	**0.006**
*Gorilla*		0.188	**<0.001**	**0.035**
*Pan*			**<0.001**	**0.006**
*Homo*				**<0.001**

*Note*: Results in bold are statistically significant (*p* <0.05).

**TABLE 7:** Pairwise Wilcoxon test on principal component 2 scores

	Gorilla	Pan	Homo	A. Afarensis
*Pongo*	**0.003**	**<0.001**	**<0.001**	**0.006**
*Gorilla*		0.102	0.523	0.662
*Pan*			**<0.001**	0.249
*Homo*				**0.014**

*Note*: Results in bold are statistically significant (*p* <0.05).

**TABLE 8:** Pairwise Wilcoxon test on principal component 3 scores

	Gorilla	Pan	Homo	A. Afarensis
*Pongo*	0.895	0.807	0.987	**<0.001**
*Gorilla*		1.000	0.987	**<0.001**
*Pan*			0.726	**0.006**
*Homo*				**<0.001**

*Note*: Results in bold are statistically significant (*p* <0.05).

### 3.4 PC1 shape and grouping

**FIGURE 5:**
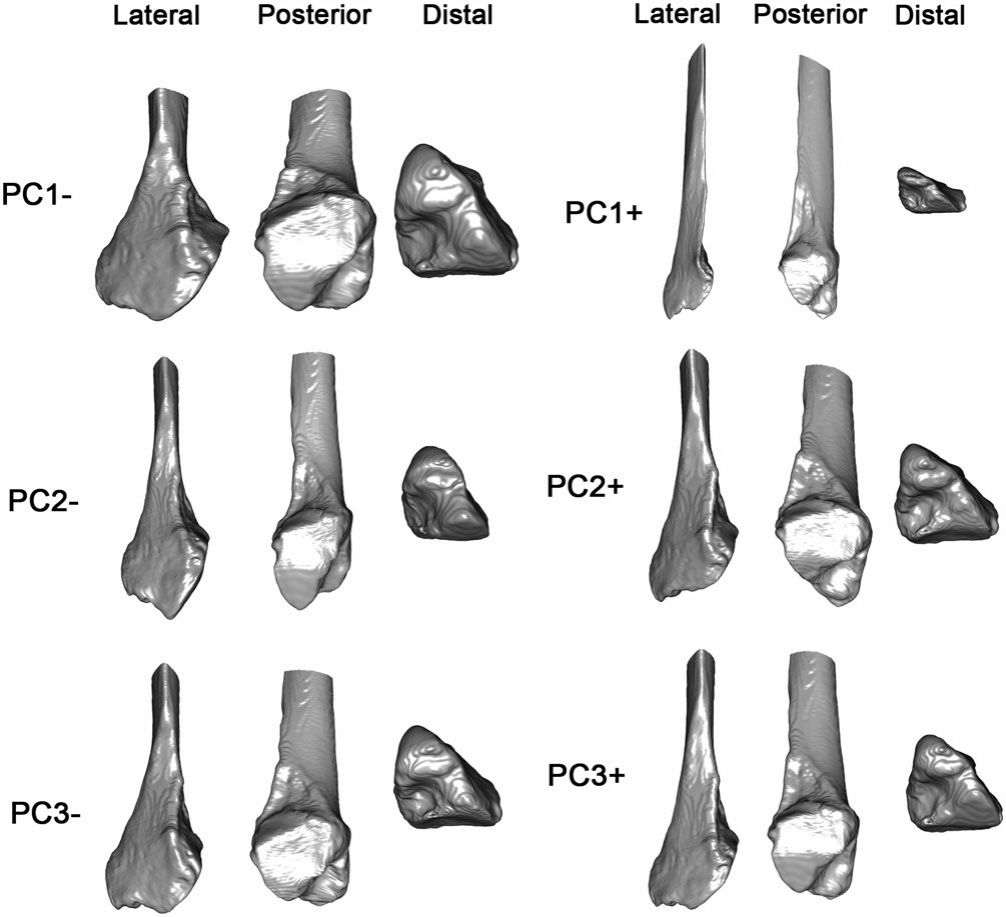
Shape changes of the right distal fibula along the first three principal components (PCs) (see text for explanation). Lateral = lateral view, medial on the right; posterior = posterior view, anterior on the left; distal = distal view, anterior on the right and medial on the bottom

The shape variations captured by the PC1 (Figure 5) are informative in distinguishing humans from great apes (SOM Figure S4). The positive side of the PC1 axis is occupied by humans, and describes a distal fibula characterized by an extremely cranially elongated STS; a proximal fibulotalar facet (PAF) almost parallel to the sagittal plane (i.e., medially facing); a cranial border of PAF perpendicular to the longitudinal axis of the fibula; a large, medially facing distal fibulotalar articular facet (DAF); a mediolaterally flattened, downward pointing and laterally facing lateral malleolus; and a narrow and shallow malleolar fossa. The negative axis of the PC1 is occupied by great apes and *A. afarensis*, and describes a distal fibula characterized by a cranially shortened STS; a larger and more downward facing PAF; a cranial border of PAF oblique to the longitudinal axis of the fibula; a smaller and more downward facing DAF; a mediolaterally thicker, laterally pointing and more anteriorly facing lateral malleolus; and a wider and deeper malleolar fossa.

### 3.5 PC2 shape and grouping

The shape variations captured by the PC2 (Figure 5) are informative in distinguishing *Pongo* from the other genera (SOM Figure S5). The positive side of the PC2, occupied by African great apes, *A. afarensis* and humans, describes a distal fibula characterized by an anteroposteriorly elongated and rectangle‐shaped (longer axis anteroposteriorly oriented) PAF; a small and downward facing DAF; a distally pointing, moderately anteriorly facing lateral malleolus,; and a narrow, relatively deep malleolar fossa whose craniocaudal axis forms an angle of about 45° with the longitudinal axis of the fibula. The negative side of PC2, occupied by *Pongo*, describes a distal fibula characterized by a craniocaudally elongated and almost quadrate in shape PAF which faces more medially; a larger, more distally elongated and more medially facing DAF; a laterally pointing and craniocaudally shortened, thicker and more anteriorly facing lateral malleolus; and a wider and shallower malleolar fossa, whose craniocaudal axis forms a steeper angle with the longitudinal axis of the fibula.

### 3.6 PC3 shape and grouping

The shape variations captured by the PC3 (Figure 5) are informative in distinguishing *A. afarensis* from the extant genera (SOM Figure S6). The positive side of the PC3 axis, occupied by great apes and humans, describes a distal fibula characterized by a cranial border of PAF almost perpendicular to the longitudinal axis of the fibula; a large DAF; a moderately thin lateral malleolus; and a wide malleolar fossa. The negative side of the PC3 axis, occupied by *A. afarensis*, describes a distal fibula shape characterized by a larger PAF; a cranial border of PAF forming a steeper angle with the longitudinal axis of the fibula; a smaller DAF; a thicker lateral malleolus; and a narrower malleolar fossa.

## 4 DISCUSSION

The aim of this paper was to describe the distal portion of the fibula in extant hominids and australopiths using a 3D‐GM approach. Our first hypothesis was that great apes would have a more anteriorly oriented lateral malleolus and more downward facing fibulotalar articular facets (proximal and distal, PAF and DAF, respectively) than humans. Our second hypothesis was that humans would have a more craniocaudally elongated STS than great apes. Results support our hypotheses and agree with previous studies performed using traditional morphometry to evaluate the distal fibula morphology of extant hominids and australopithecines (Marchi, 2015b; Stern & Susman, 1983) further highlighting the importance of the fibula to understand the amount of arboreality present in fossil hominins. Moreover, the use of the 3D‐GM method allowed us to point out differences in the distal fibula morphology of humans and great apes that were not possible to discern using traditional morphometry, such as the importance of the STS in separating the different groups on the basis of locomotion, the thicker lateral malleolus in great apes than in humans implying a wider peroneal groove in the formers, and a deeper and wider malleolar fossa in great apes than in humans. These new anatomical evidence on the distal fibula may be useful for a better understanding of the locomotor adaptations of *A. afarensis*.

### 4.1 Extant hominids

The 3D‐GM analyses performed in this research pointed out quantitatively the importance of the STS in separating humans and great apes, with the former characterized by a craniocaudally elongated STS (see Figures 1 and 5). Based on the differences in the anatomy of the mm. *peroneus longus* and *peroneus brevis* between humans and great apes outlined above, we suggest that a craniocaudally elongated STS can be used as evidence of terrestrial bipedality within hominids. While some difference in the craniocaudal length of STS among great apes in present (see Figure 1), they are all grouped along PC1 (Figure 4) and clearly separated from humans indicating distinct morphologies for the two extant groups.

Stronger, more accurate and precise range of motion in inversion/eversion—and plantarflexion—of the foot is required by great apes than humans because of the climbing behavior they are involved in when moving in trees (DeSilva, 2009; Holowka et al., 2017). As for humans, their obligate terrestrial bipedal locomotion does not require powerful inversion/eversion of the foot given that they move tendentially on a flat substrate. Stronger and more precise movements are generally accomplished in mammals through long fibers—and short tendons—in their muscle tendon units (Biewener, 2016). Therefore, the longer muscle bellies—and shorter tendons—of chimps' peroneal muscles than humans' can be explained by the powerful inversion/eversion movements customarily carried out by great apes when moving in the arboreal environment where they live. We suggest that the craniocaudally elongated STS observed in humans is associated to their shorter peroneal muscle bellies and longer peroneal tendons compared to great apes, which agree with the different ankle kinematics of the two groups. Studies aimed at describing the covariation between leg muscular (Marchi et al., 2018) and bone morphology in relationship to climbing behavior in great apes and humans are necessary to further test this hypothesis.

In agreement with previous studies, the 3D‐GM analysis pointed out a relatively more anteriorly facing lateral malleolus in great apes than in humans correlated with more developed peroneal muscles (Stern & Susman, 1983). Moreover, great apes show an oblique superior margin of PAF which has previously been associated with a plantarflexion set of the ankle by Stern and Susman (1983), and its presence in great apes would indicate their greater ankle joint mobility compared to humans. The results of the present study also quantitatively demonstrated (as previously noted by Stern & Susman, 1983) that both the proximal and distal fibulotalar articular facets are more medially facing in humans and more downward facing in great apes, in agreement with the general greater vertical component of the ground reaction force transmitted by the fibula through the talus expected in the great ape ankle joint compared to humans. This finding is supported by theoretical work on the loading patterns in the ankle joint (Preuschoft, 1970) and empirical studies conducted on the fibula/tibia diaphyseal robusticity (Marchi, 2007, 2015a; Marchi et al., 2019), as well as anatomical studies showing greater dorsiflexion and eversion of the ankle joint in the arboreal setting (Barnett & Napier, 1953; DeSilva, 2009). Experimental studies (i.e., kinetic and kinematic) are necessary to precisely quantify the amount of vertical force to which the distal fibular articular joint is subjected to in the arboreal environment in great apes.

The 3D‐GM analysis also pointed out the presence of a thicker lateral malleolus in great apes than in humans, implying a wider peroneal groove in the formers (see Figure 5). The enlarged peroneal groove found in great apes may be related to the observed greater mass of peroneal muscles in great apes compared to humans (e.g., Stern & Susman, 1983; Mclean & Marzke, 1994; Aiello & Dean, 2002; Payne et al., 2006). Though the relationship between tendons and muscles can be quite complicated, across mammals, muscle mass and tendon size (generally) scale isometrically with body size (Pollock & Shadwick, 1994). However, the specific relationship between a muscle and its tendon can change depending on several factors, including: a. the identity of the muscle and tendon; b. the species; c. the animal's exercise/loading history (Kubo et al., 2010); and d. age (Mersmann et al., 2015; Narici & Maganaris, 2006; Stenroth et al., 2012). However, when these variables are all the same and activity level is controlled for, studies suggest that tendon size can be used to predict muscle size (An et al., 1991; Germiller et al., 1998; Seynnes et al., 2009). Therefore, it is reasonable to suggest that the larger personal groove found in great apes may be associated to the observed larger peroneal muscles observed in great apes than humans.

EMG studies found that peroneal muscles are active during the support phase of locomotion on both humans and great apes (Stern & Susman, 1983; Jungers, Meldrum & Stern, 1993). However, in humans they are much more active when walking on a medial incline, therefore when everting the inverted foot (Bavdek et al., 2018). This is the way peroneal muscles are active during vertical climbing in great apes, therefore it may be suggested that those muscles are more active in great apes than in humans. However, EMG studies performed so far on peroneal muscles have not been done on the same behaviors for humans and great apes. That is, EMG studies are missing of humans climbing and on chimpanzee involved in bipedal locomotion: such studies are necessary to further test this hypothesis.

A novel find of the 3D‐GM analysis is the presence of a deeper and wider malleolar fossa in great apes than in humans. In humans, the malleolar fossa is the lateral insertion of the posterior tibiofibular and the posterior talofibular ligaments (Ebraheim et al., 2006; Golanó et al., 2010; Hermans et al., 2010; Perrich et al., 2009), which together with the anterior talofibular, calcaneofibular and the deltoid ligaments control and limit the mobility of the ankle joint (Kleipool & Blankevoort, 2010). To our best knowledge there are no data on the function of these ligaments in great apes. In humans, the posterior tibiofibular ligament is a thick ligament whose function is not yet completely understood, it being proposed to be a stabilizer of the talocrural joint stability, to prevent posterior talar translation, or to prevent internal rotation forces in the ankle joint (Ebraheim et al., 2006; Taylor et al., 1992). On the other hand, it has been observed that the posterior talofibular ligament is relaxed in the neutral ankle position and in plantarflexion, while in dorsiflexion the ligament is tensed. We suggest that the observed deeper and wider malleolar fossa of great apes compared to humans might be the consequence of a larger talofibular ligament in the former, possibly consequence of the higher frequency and magnitude of dorsiflexion experienced during climbing behavior by great apes (DeSilva, 2009). Additional anatomical and biomechanical studies on the human and great ape ankle ligaments are necessary to test this hypothesis.

Within great apes, the 3D‐GM analysis of the distal fibula shows a separation between African great apes and *Pongo*. The distal fibula of *Pongo* is characterized by proximal and distal fibulotalar articular facets which are more medially facing than in African great apes (see Figure 5, PC2). According to what was discussed above, it would mean that in *Pongo* a lower amount of vertical force is transmitted through the talus than in African great apes.

While *Pan* and *Gorilla* differ in the amount of time spent in the trees, they are both quadrupedal and spend much time on the ground (Doran, 1996; Remis, 1995, 1998) where they employ the characteristic knuckle‐walking locomotion (Tuttle, 1967). *Pongo* is the largest mammal that travels regularly in the forest canopy, and its suspensory capacities have been essential in permitting the evolution and maintenance of its great body size in a habitat with tapering branches and frequent gaps (Cant, 1987). *Pongo* shows different locomotor repertoires when traveling in the canopy, such as quadrupedalism, suspension, clambering and vertical climbing/descending (e.g., Sugardjito & van Hoof, 1986; Cant, 1987; Thorpe & Crompton, 2005; Manduell et al., 2011; Manduell et al., 2012). One of the main differences between *Pongo* and the African great apes is that the former often moves on flexible supports (Manduell et al., 2012; Thorpe & Crompton, 2006). It is reasonable to think that in *Pongo*, which moves on flexible supports, a lower vertical component of the ground reaction force is transmitted through the fibula to the talus than in African great apes, which do not habitually move on such flexible supports. Following the argument exposed above for the human‐great ape comparison, this may be the reason for the generally more medially oriented fibulotalar articular facets of *Pongo* compared to African great apes.

The use of flexible supports by *Pongo* while traveling in trees has been demonstrated to lower the energetic cost of their locomotion (Thorpe et al., 2006) but, to our best knowledge, no experimental studies have so far investigated the vertical component of the ground reaction forces to which the ankle joint of *Pongo* is subjected during arboreal locomotion on flexible substrates. Moreover, while it is well known that primates use more compliant walking in arboreal environment than in terrestrial environment (Larney & Larson, 2004; Schmitt, 1999), there are no comparative data on the forces developed at the ankle joint in *Pongo* and African great apes. Experimental data on vertical forces developed at the ankle joint, and in particular at the fibulotalar articulation, in *Pongo* and African great apes are needed to further test the hypothesis that the observed more medially facing fibulotalar articular facets of *Pongo* are correlated with lower vertical components of the ground reaction force to which their ankle joint is subjected when they move on more flexible substrate compared to African great apes.

### 4.2 Australopithecus afarensis

*Australopithecus afarensis* shows a mosaic of primitive (ape‐like) and derived (human‐like) traits which may have allowed the species to be both arboreal and terrestrial. Retained primitive traits such as relatively long and curved phalanges in the hand and foot, and other traits in the lower limb and foot testify their ability in climbing and suspension (Stern & Susman, 1983; Duncan et al., 1994; Stern, 2000; Richmond, 2003; Alemseged et al., 2006; Green & Alemseged, 2012; Churchill et al., 2013). On the other hand, derived traits in the pelvis, the knee and the foot testify about their effective terrestrial bipedal locomotion (Alemseged et al., 2006; DeSilva, 2009; DeSilva et al., 2019; Haile‐Selassie et al., 2010; Kimbel & Delezene, 2009; Latimer et al., 1987; Latimer & Lovejoy, 1990a, 1990b; Ward et al., 2012; White, 1980). A previous study performed on the distal fibular morphology of *A. afarensis*, provided partial support to the hypothesis that *A. afarensis* was a terrestrial biped involved in climbing behavior (Marchi, 2015b).

The results of the 3D‐GM investigation of the distal fibular morphology further support previous results obtained using traditional morphometry (Marchi, 2015b; Stern & Susman, 1983). The Hadar hominins appear to cluster separately from all extant genera and closer to the African great apes than to humans. The shorter STS of *A. afarensis* compared to humans would suggest for the former longer peroneal muscle bellies and shorter tendons than humans, which have been proposed to be associated with climbing behavior (Stern & Susman, 1983). The present study also finds in *A. afarensis* other traits in the distal fibula that are generally associated more to climbing behavior and less to bipedalism, such as a more anteriorly facing, laterally pointing lateral malleolus and deeper and larger malleolar fossa.

What appears from this study is that the morphology of the distal fibula of *A. afarensis*, although different from all living hominids, is closer to African great apes than to humans. As already proposed (Marchi, 2015b) two reasons may be responsible of this result. The first may be that the unique anatomy of *A. afarensis* reflects the not complete humanlike bipedal adaptations of the species and therefore the primitive retentions in the distal fibula would be less functionally restrictive for bipedality than other section of the body like for example the distal tibia (DeSilva, 2009). Alternatively, the mosaic of traits observed in the lower limb of *A. afarensis* (Kimbel & Delezene, 2009) may imply a unique way of loading of the distal leg in the Hadar hominins, different from what observed in all living hominids (Marchi, 2007). More *A. afarensis* distal fibulae, and in particular associated tibiae and fibulae, should be found and investigated to test the two above hypotheses.

A limitation of the present work may be the limited sample size for extant great apes. Though many long bone laser and CT scans are now freely available on the web, the fibula is rarely included among scanned bones. Moreover, when the fibula is present, due to its small size compared to the other long bones, scan definitions are usually not good enough to point out the anatomical characteristics where to locate the landmarks used in the 3D‐GM analysis. Despite this potential limitation, the segregation among extant species is always clear and functionally interpretable in every analysis presented here, making this new method for predicting the degree of arboreality from distal fibular morphology a valuable addition to the ones already present in literature. In future we plan to collect more fibula laser/CT scans of extant hominoids and other primates to further test the reliability of the method presented here.

## 5 CONCLUSION

The fibula is a scarcely investigated bone in paleoanthropology although recent studies have highlighted its importance to understanding locomotor adaptations in extant hominids and fossil hominins. The results of the 3D‐GM study of the distal fibular morphology shows that extant hominids more frequently involved in climbing behavior (i.e., great apes) are characterized by more downward facing fibulotalar articular facets, more anteriorly facing lateral malleolus and wider and deeper malleolar fossa than humans. Within great apes, *Pongo* is characterized by more medially facing fibulotalar articular facets possibly due to the lower vertical component of the ground reaction force to which the distal fibula and the talus are subjected in the ankle joint of this species (which habitually move on flexible supports) than in African great apes. The results of the application of the 3D‐GM method to the Hadar hominins support previous analyses which highlighted a distal fibula morphologically different from any extant hominid and with several primitive retentions suggesting a climbing component in the locomotor repertoire of *A. afarensis*.

### AKCNOWLEDGEMENTS

The authors would like to thank the curators at the various museums and institutions where comparative data were collected: B. Zipfel and B. Billings (University of the Witwatersrand, South Africa), K. Isler and E. Langenegger (University of Zurich, Irchel, Switzerland), G. Grupe and O. Röhrer‐Ertl (University of Munich, Germany). We would like also to thank C. Turcotte (New York University) and A. Hartstone‐Rose (North Carolina State University) for the useful suggestions concerning muscle size‐tendon size relationships. D.G.‐M. and M.B. are funded by project CGL2015‐63648‐P (MINECO; Spain) and by project PID2020‐115854GB‐I00, MCIN/AEI/10.13039/501100011033. Open Access Funding provided by Universita degli Studi di Pisa within the CRUI‐CARE Agreement.

## CONFLICT OF INTEREST

The authors declare no conflict of interest.

## AUTHOR CONTRIBUTIONS

**Damiano Marchi:** Conceptualization (lead); data curation (lead); funding acquisition (lead); investigation (equal); project administration (lead); resources (lead); supervision (lead); validation (equal); visualization (supporting); writing – original draft (lead); writing – review and editing (equal). **Andreas Rimoldi:** Formal analysis (lead); investigation (equal); methodology (equal); software (equal); validation (equal); visualization (lead); writing – original draft (supporting); writing – review and editing (equal). **Daniel García‐Martínez:** Methodology (equal); software (equal); validation (equal); writing – review and editing (equal). **Markus Bastir:** Methodology (equal); software (equal); validation (equal); writing – review and editing (equal).

## Supporting information


**Appendix S1**: Supporting information

## Data Availability

The data that support the findings of this study are available upon reasonable request.
