# Supplementary material for: Morphological correlates of distal fibular morphology with locomotion in great apes, humans, and Australopithecus afarensis
Source: Am J Biol Anthropol. 2022 Mar 17;178(2):286–300. doi: 10.1002/ajpa.24507 (PMC9314891; doi:10.1002/ajpa.24507)
Supplement: Supplementary file 1 — Appendix S1: Supporting information [file AJPA-178-286-s001.pdf]

Supplementary Material

(Long Title): Morphological correlates of distal fibular morphology with locomotion in great apes, humans and *Australopithecus afarensis*

(Short Title): Distal fibula morphology in hominids

Damiano Marchi[1,2,*], Andreas Rimoldi[1], Daniel Garcia-Martinez[3,4,2], Markus Bastir[4,2]

[1] *Department of Biology, University of Pisa, via Derna 1, Pisa, 56126, Italy*

[2] Centre for the Exploration of the Deep Human Journey, *University of the Witwatersrand, Private Bag 3, Wits 2050, South Africa.*

[3] *Centro Nacional de Investigación sobre la Evolución Humana (CENIEH). Paseo Sierra de Atapuerca 3, 09002 Burgos, Spain*

[4] *Paleoanthropology group, Museo Nacional de Ciencias Naturales (MNCN-CSIC), J.G. Abascal 6, 28006 Madrid, Spain*

***Correspondence**

E-mail address: damiano.marchi@unipi.it (D. Marchi)

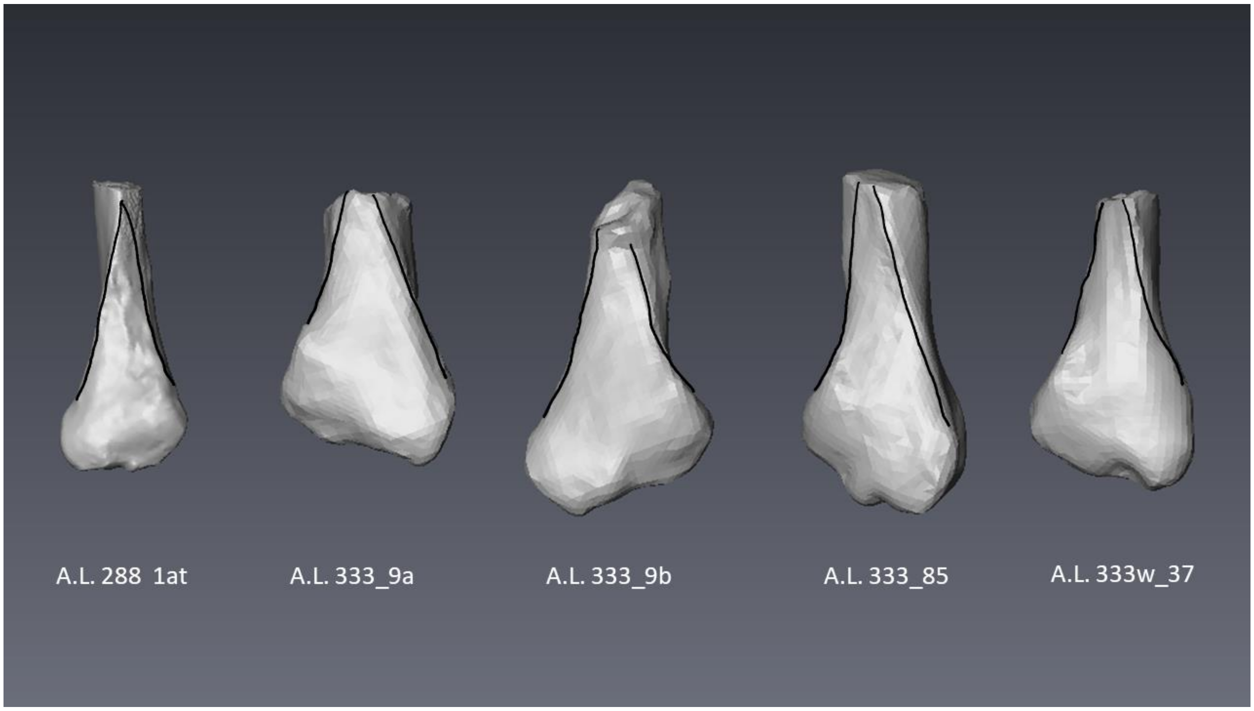

Figure S1: Three-dimensional rendering of the five Australopith distal fibulae. Black curves outline the anterior and posterior borders of the subcutaneous triangular surface: note that the surface is completely preserved only in A.L. 288-1at. However, using the landmark configuration shown in Fig. 2 of the main text, only the fixed landmark 1 had to be estimated out of 71 landmarks present in each configuration.

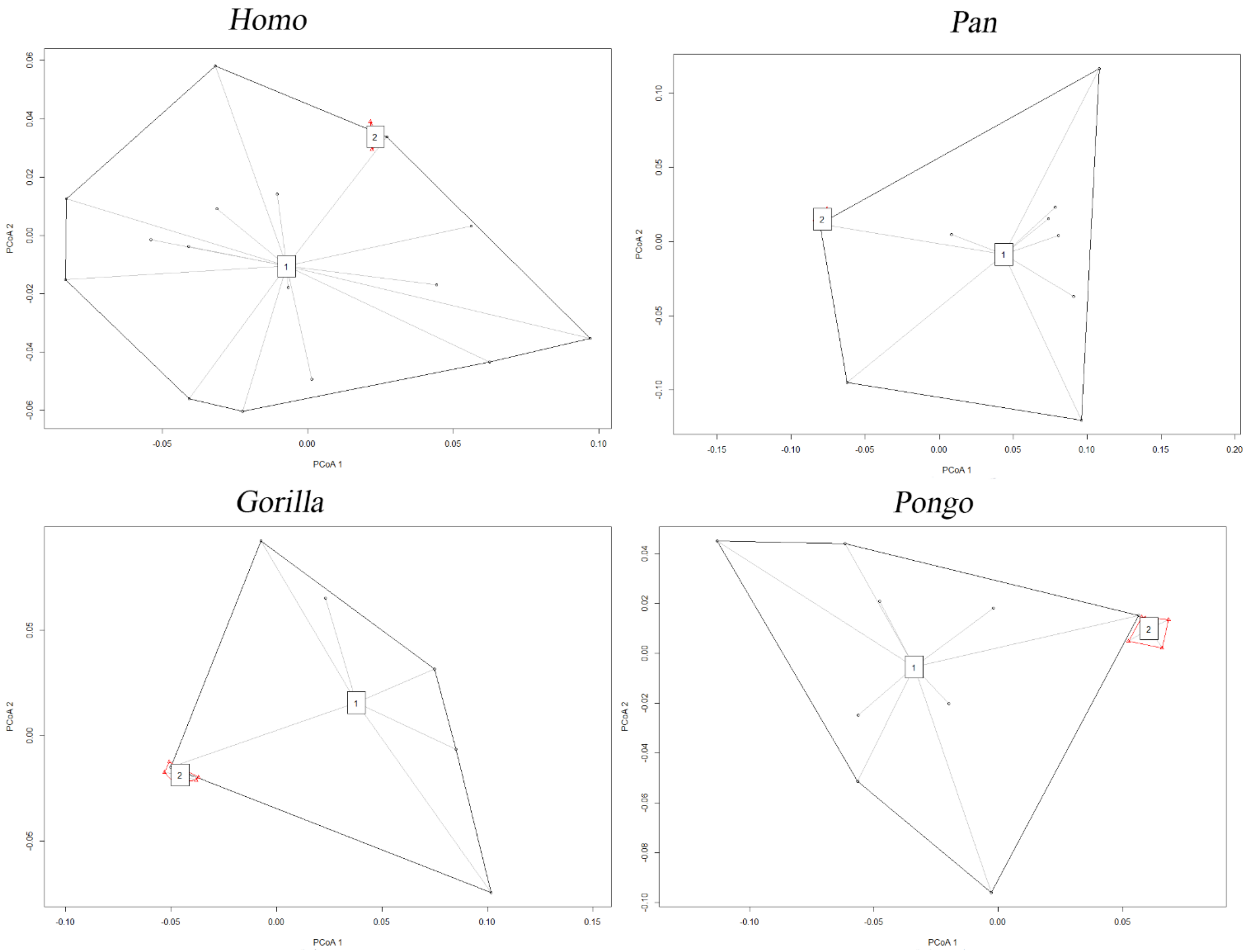

Figure S2. Scatterplot of PC1 vs PC2 scores for *Homo*, *Pan*, *Gorilla* and *Pongo* as resulted from the repeatability test: black circles are single individuals, labelled “1”, red triangles are repeated measures, labelled 2.

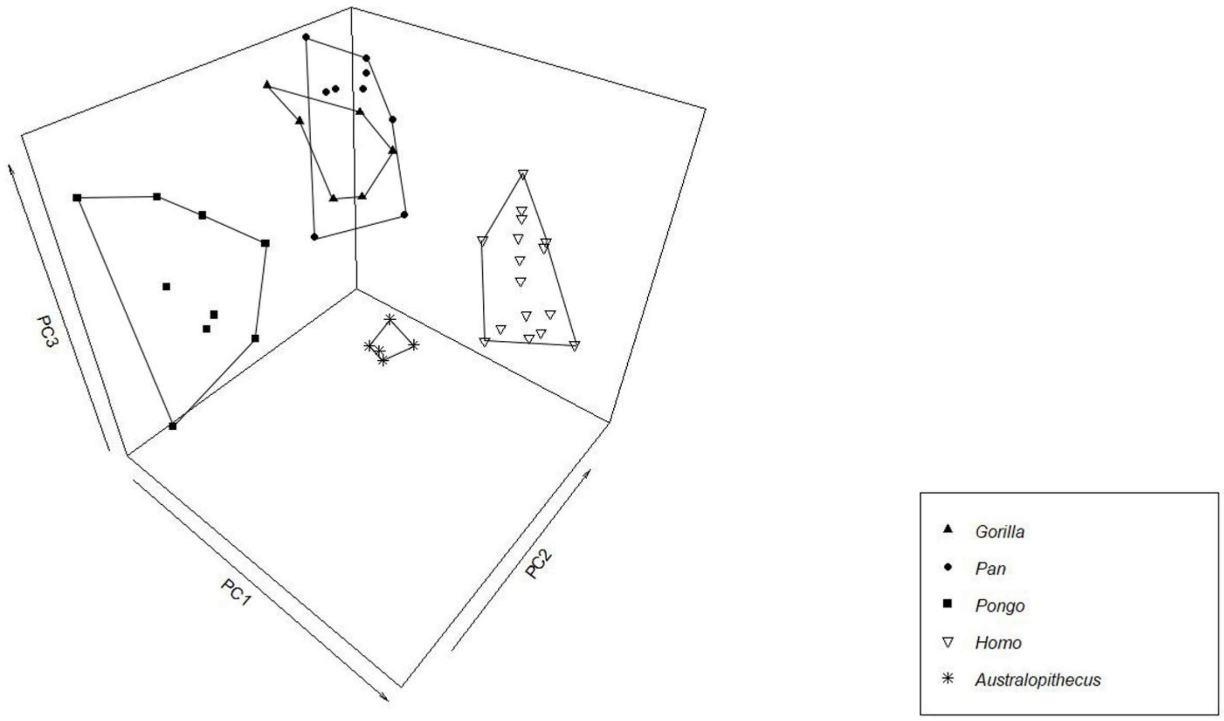

Figure S3: Scatterplot of first, second and third principal components (PC1, PC2 and PC3, respectively) scores of extant samples (*Homo*, *Pan*, *Gorilla* and *Pongo*) and fossil specimens A.L. 288-1at, A.L. 333-9a, A.L. 333-9b, A.L. 333-85, A.L. 333w-37 (labeled '*Australopithecus'* in figure).

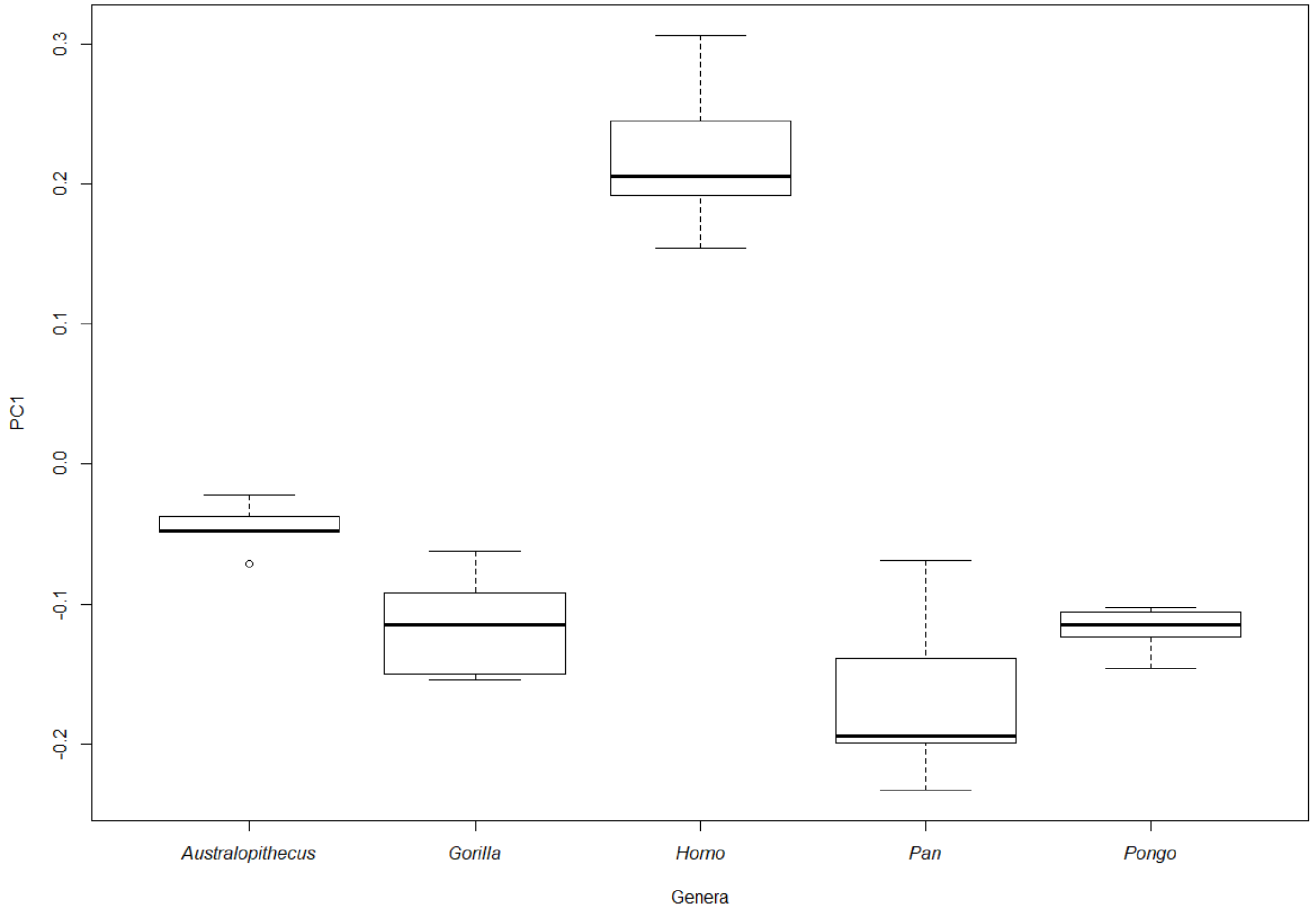

Figure S4: Boxplot of the first principal component (PC1) scores for *Homo*, *Pan*, *Gorilla* and *Pongo* compared to *Australopithecus afarensis* (specimens A.L. 288-1at, A.L. 333-9a, A.L. 333-9b, A.L. 333-85 and A.L. 333w-37) labeled "*Australopithecus*". Black lines are the medians, boxes represent the interquartile ranges, whiskers the non-outliers range and empty circles the outliers.

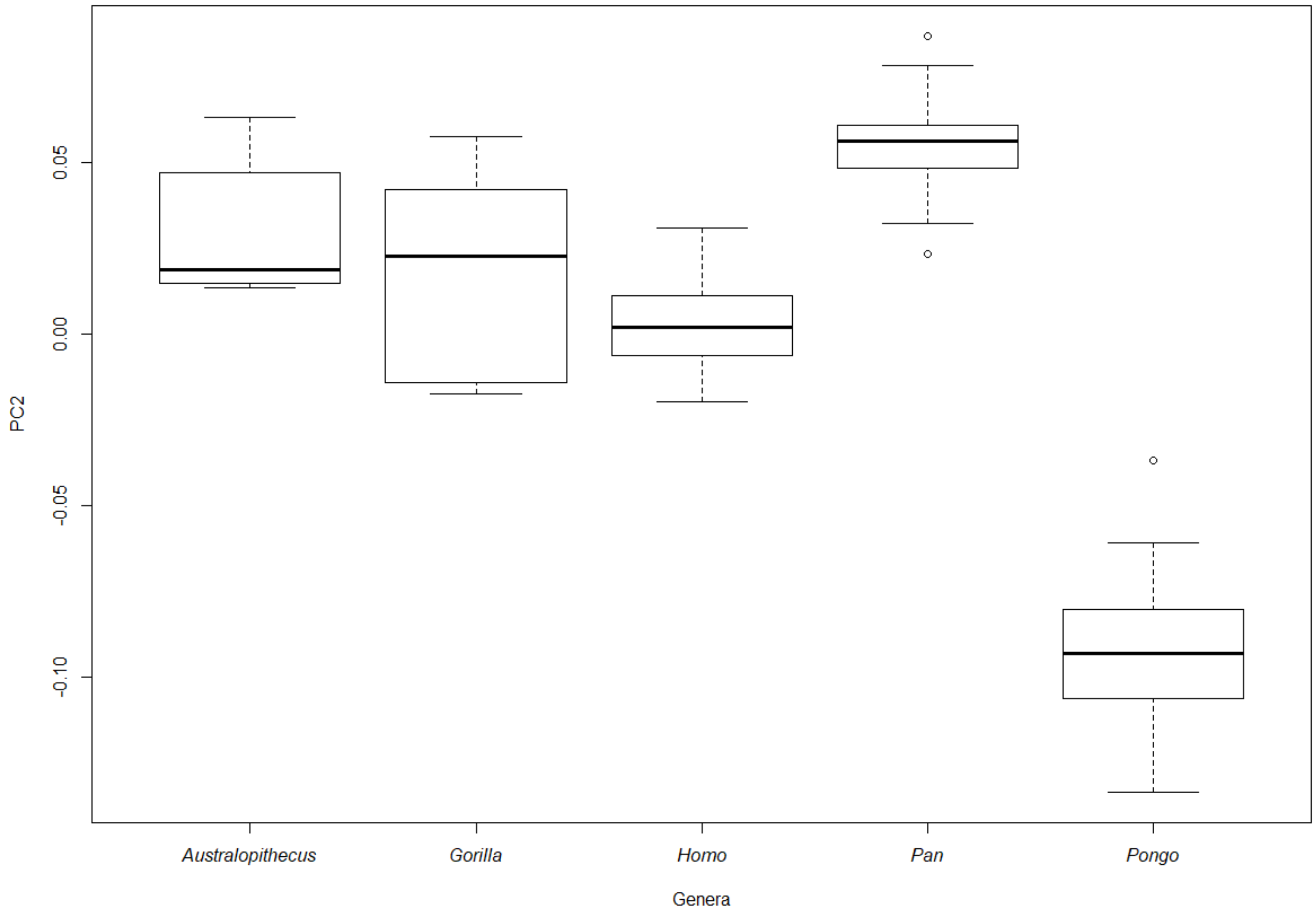

Figure S5: Boxplot of the first principal component (PC2) scores for *Homo*, *Pan*, *Gorilla* and *Pongo* compared to *Australopithecus afarensis* (specimens A.L. 288-1at, A.L. 333-9a, A.L. 333-9b, A.L. 333-85 and A.L. 333w-37) labeled "*Australopithecus*". Black lines the medians, boxes represent the interquartile ranges, whiskers the non-outliers range and empty circles the outliers.

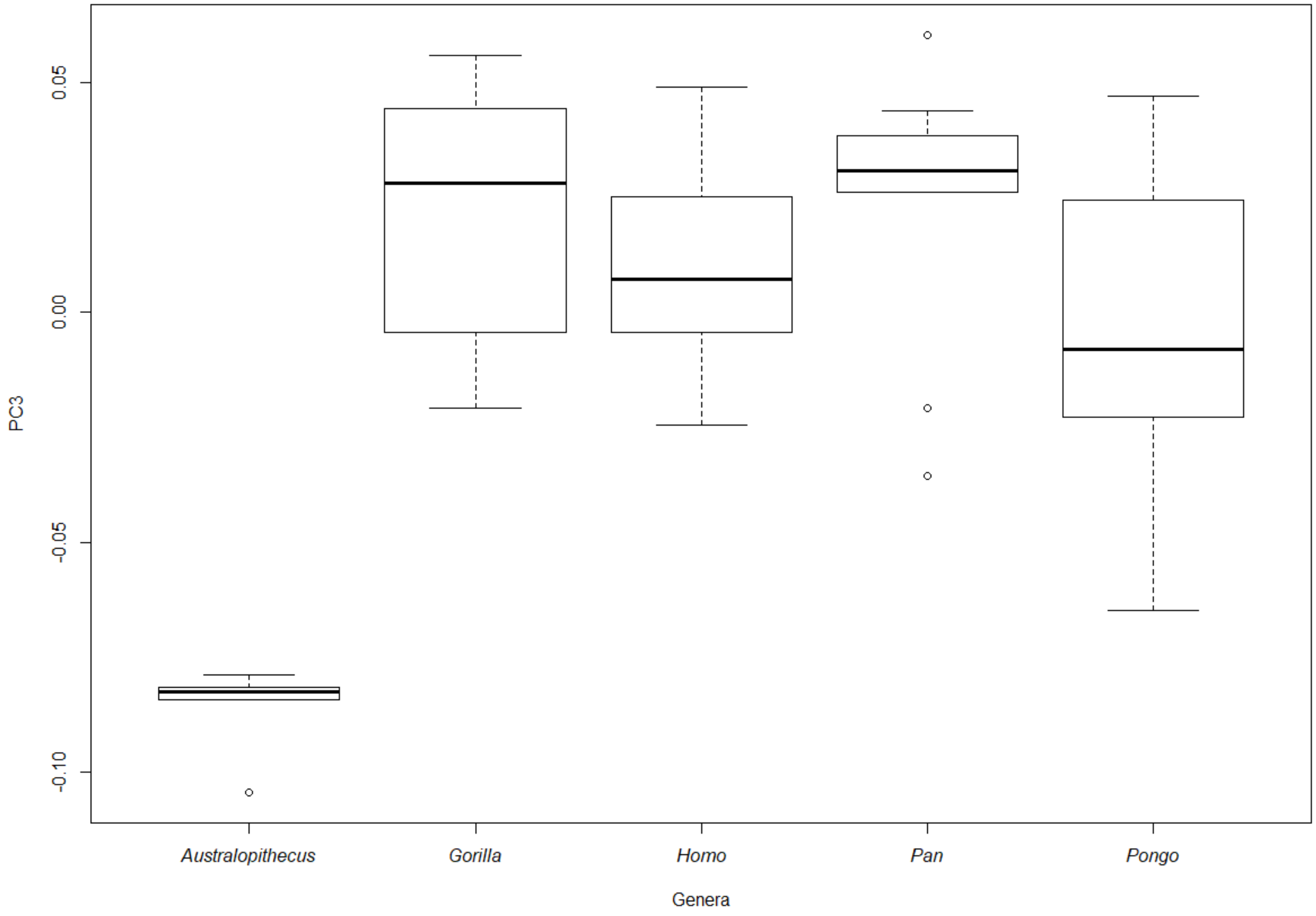

Figure S6: Boxplot of the first principal component (PC3) scores for *Homo*, *Pan*, *Gorilla* and *Pongo* compared to *Australopithecus afarensis* (specimens A.L. 288-1at, A.L. 333-9a, A.L. 333-9b, A.L. 333-85 and A.L. 333w-37) labeled "*Australopithecus*". Black lines the medians, boxes represent the interquartile ranges, whiskers the non-outliers range and empty circles the outliers.
